# Outcomes of modified pneumatic retinopexy with transscleral subretinal fluid drainage for primary rhegmatogenous retinal detachment

**DOI:** 10.1186/s40662-026-00506-z

**Published:** 2026-08-26

**Authors:** Qintuo Pan, Renwan Liu, Huirong Pan, Meiqi Liu, Jiayi Wei, Saifei Zhang, Xuting Hu, Zhaoliang Zhang, Wei Chen, Zongduan Zhang

**Affiliations:** National Clinical Research Center for Ocular Diseases, Eye Hospital, Wenzhou Medical University, Wenzhou, 325027 China

**Keywords:** Modified pneumatic retinopexy, Rhegmatogenous retinal detachment, Transscleral subretinal fluid drainage, Air, Laser photocoagulation

## Abstract

**Background:**

Primary rhegmatogenous retinal detachment (RRD) occurs when a retinal tear or break causes fluid from inside the eye to seep behind the retina, which pushes it away from the wall of the eye. This can cause permanent vision loss if not treated. Which of three current interventions is optimal to treat RRD has not been established. We evaluated the anatomical and visual outcomes of a modified pneumatic retinopexy (mPnR) that drains fluid from beneath the retina to treat RRD.

**Methods:**

This retrospective single-center case series included 53 consecutive eyes of 53 patients with primary RRD who met the pneumatic retinopexy versus vitrectomy trial criteria. All patients underwent mPnR that comprised transscleral subretinal fluid drainage, intravitreal air injection, and postoperative laser photocoagulation of retinal breaks. The main outcome measures were single-surgery anatomical success rates and best-corrected visual acuity (BCVA) at 3 months postoperatively.

**Results:**

The single-surgery anatomical success rate was 51 of 53 (96.23%). Three months postoperatively, the median BCVA had significantly improved from a preoperative logarithm of the minimum angle of resolution (logMAR) of 0.10 (interquartile range [IQR], 0.00–0.60) to 0.02 (IQR, 0.00–0.20) (*P* < 0.001). The anatomic success rate was 100% for the macula-off subgroup (n = 20), as BCVA improved from 0.75 (IQR, 0.43–1.18) to 0.20 (IQR, 0.04–0.30) logMAR *(P* < 0.001). Two eyes (3.77%) with recurrent detachment and one eye (1.89%) with a secondary macular epiretinal membrane required subsequent pars plana vitrectomy.

**Conclusions:**

The mPnR procedure is efficient and minimally invasive, expansile gas is not required, and preliminary safety and efficacy profiles were favorable. Therefore, mPnR offers a simplified, feasible surgical option for treating eligible patients with primary RRD.

**Supplementary Information:**

The online version contains supplementary material available at 10.1186/s40662-026-00506-z.

## Background

Rhegmatogenous retinal detachment (RRD), the most common form of retinal detachment, occurs when liquefied vitreous passes through a full-thickness retinal break, resulting in the neurosensory retina separating from the underlying retinal pigment epithelium [1]. Risk factors for RRD include posterior vitreous detachment, cataract surgery, myopia, degenerative retinoschisis, lattice degeneration, and ocular trauma [2]. The incidence of RRD ranges from 6.3 to 17.9 cases per 100,000 population, and it increases with age [3]. Current primary surgical treatments for RRD include pars plana vitrectomy (PPV), scleral buckling (SB), and pneumatic retinopexy (PnR). However, the optimal intervention remains a matter of ongoing debate [4].

Both PPV and SB have been the preferred surgical approaches for RRD for several decades due to favorable single-surgery anatomic success rates [4–6]. Initially implemented by Domínguez as an outpatient procedure for RRD repair without conjunctival incision [7], PnR was later popularized by Hilton and Grizzard [8]. A prospective, multicenter, randomized controlled trial that compared PnR with SB found no significant difference in single-surgery reattachment rates, but visual acuity was better at 6 months and 2 years after PnR [9, 10]. Furthermore, PnR provides superior functional and anatomic outcomes relative to PPV and with equivalent primary reattachment rates in selected patients with RRD [11–13]. Although PnR offers advantages such as procedural efficiency, minimal invasiveness, and reduced resource demand compared with PPV or SB, which typically require an operating room, its conventional approach requires strict and prolonged postoperative positioning for at least 5 days and up to 3 weeks to allow subretinal fluid (SRF) resorption [6]. Furthermore, an intravitreal gas bubble can cause sustained visual impairment for several weeks if supplemental gas injections are required [14].

Consequently, we modified the conventional PnR technique by incorporating transscleral drainage into SRF. This facilitated rapid postoperative retinal reattachment, eliminated the need for intraocular expansile gas, and preserved the inherent advantages of PnR. We evaluated the primary anatomical success rates and visual outcomes of the modified pneumatic retinopexy (mPnR) to treat primary RRD.

## Methods

This single-center retrospective cohort study was approved by the Ethics Committee of the Eye Hospital of Wenzhou Medical University (Approval No: 2025-043-X-02-01). All procedures adhered to the tenets of the Declaration of Helsinki.

We reviewed the medical records of consecutive patients with primary RRD who presented to the Eye Hospital at Wenzhou Medical University between March 19, 2025, and October 5, 2025, and were treated using mPnR. We then selected only eyes with a minimum postoperative follow-up interval of 3 months.

The inclusion criteria for RRD were based on the Pneumatic Retinopexy versus Vitrectomy for the Management of Primary Rhegmatogenous Retinal Detachment (PIVOT) trial [11] as follows: one or more retinal breaks confined to a 30° sector (≤ 1 clock hour); all breaks located between the 8 and 4 o’clock meridians superiorly. The exclusion criteria were clinically significant proliferative vitreoretinopathy, history of retinal detachment repair in the same eye except for previous PnR, inability to comply with postoperative positioning requirements (such as severe physical disability); eyes with a shallow SRF and extremely limited extent of retinal detachment (such as located at only 1 to 2 o’clock to the peripheral retina), and inadequate visualization of the peripheral retina or inability to implement laser photocoagulation due to media opacity. The primary outcome measures were the single-surgery anatomical success rates of mPnR and the best-corrected visual acuity (BCVA) at 3 months postoperatively.

The following clinical data were collected from the medical records of the patients: age, sex, affected eye, axial length, history of hypertension, history of diabetes, duration of RRD symptoms, preoperative and postoperative BCVA, lens status, surgical, complications, follow-up duration, wide-field fundus photography (Optos Inc., Marlborough, MA, USA), and spectral-domain optical coherence tomography (SD-OCT) using a Spectralis OCT confocal scanning laser ophthalmoscope (Heidelberg Engineering GmbH, Heidelberg, Germany). Symptom duration was defined as the interval between the onset of visual loss or field defects reported by the patient, and the date of surgery.

### Surgical technique

The pupils were adequately dilated, then the fundus was preoperatively examined. All retinal breaks and areas of lattice degeneration in the attached retina were surrounded by laser photocoagulation.

The eye surface was preoperatively anesthetized using 15 mL (75 mg) of topical proparacaine hydrochloride (National Drug Approval Number: HJ20160133) eye drops (Alcon NV, Mechelen, Belgium). The subconjunctiva was anesthetized over the quadrant corresponding to retinal breaks using 100 mg of 2% lidocaine hydrochloride (National Drug Approval Number: R25041403) (Hunan Kelun Pharmaceutical Co., Ltd., Hunan City, China). The rectus muscles adjacent to breaks were fixed transconjunctivally using traction sutures. The sclera was exposed via a localized conjunctival incision (~ 1 cm). The SRF was then drained transsclerally using a 22-gauge needle attached to a 5-mL syringe at the site of maximal retinal elevation adjacent to retinal break(s). Bevel incisions are recommended to enhance the self-sealing properties of scleral wounds. Gentle indentation with a sterile cotton swab was simultaneously applied to maintain normal intraocular pressure. A 30-gauge needle was subsequently inserted 4 mm posterior to the limbus, and filtered air was injected into the vitreous cavity during which the indentation was gradually released by digital palpation until intraocular pressure reached approximately Tn to T+1 (normal to slightly elevated). The sequence of SRF drainage and air injection was repeated to maximize SRF evacuation, while preserving the largest possible intravitreal air volume. We repeated the SRF drainage by creating a gentle scleral indentation immediately adjacent to the scleral incision with microforceps suffices to avoid repeated needle entry. The conjunctival incision was closed by electrocoagulation or suturing (Additional File 2 Supplementary Video).

Postoperatively, the patients were instructed to maintain a position with the uppermost retinal break(s) for 3–5 days to ensure continuous air tamponade. Retinal break(s) were laser photocoagulated 12–24 h postoperative, and additional treatment was applied as required.

Levofloxacin 0.5% ophthalmic solution was administered four times daily for 5 days postoperatively as infection prophylaxis. One vitreoretinal surgeon (QTP) conducted all surgical and laser procedures. The patients were followed up at 1 day, 3–7 days, 1 month, 3 months, and 6 months postoperatively.

### Statistical analysis

Data were statistically analyzed using SPSS version 27.0 (IBM Corp., Armonk, NY, USA). The BCVA values were converted to the logarithms of the minimum angle of resolution (logMAR) before analysis. Continuous variables and non-normally distributed data are presented as means ± standard deviation (SD) and as medians with interquartile ranges (IQRs). Categorical variables are expressed as frequencies and ratios (%). Continuous variables between groups were compared using independent-samples t-tests or Mann–Whitney U tests, as appropriate. Paired comparisons such as preoperative vs. postoperative BCVA were analyzed using paired t-tests or Wilcoxon signed-rank tests based on data normality. Categorical variables were compared using chi-squared tests. Values with *P* < 0.05 were considered to be statistically significant.

## Results

**Fig. 1:**
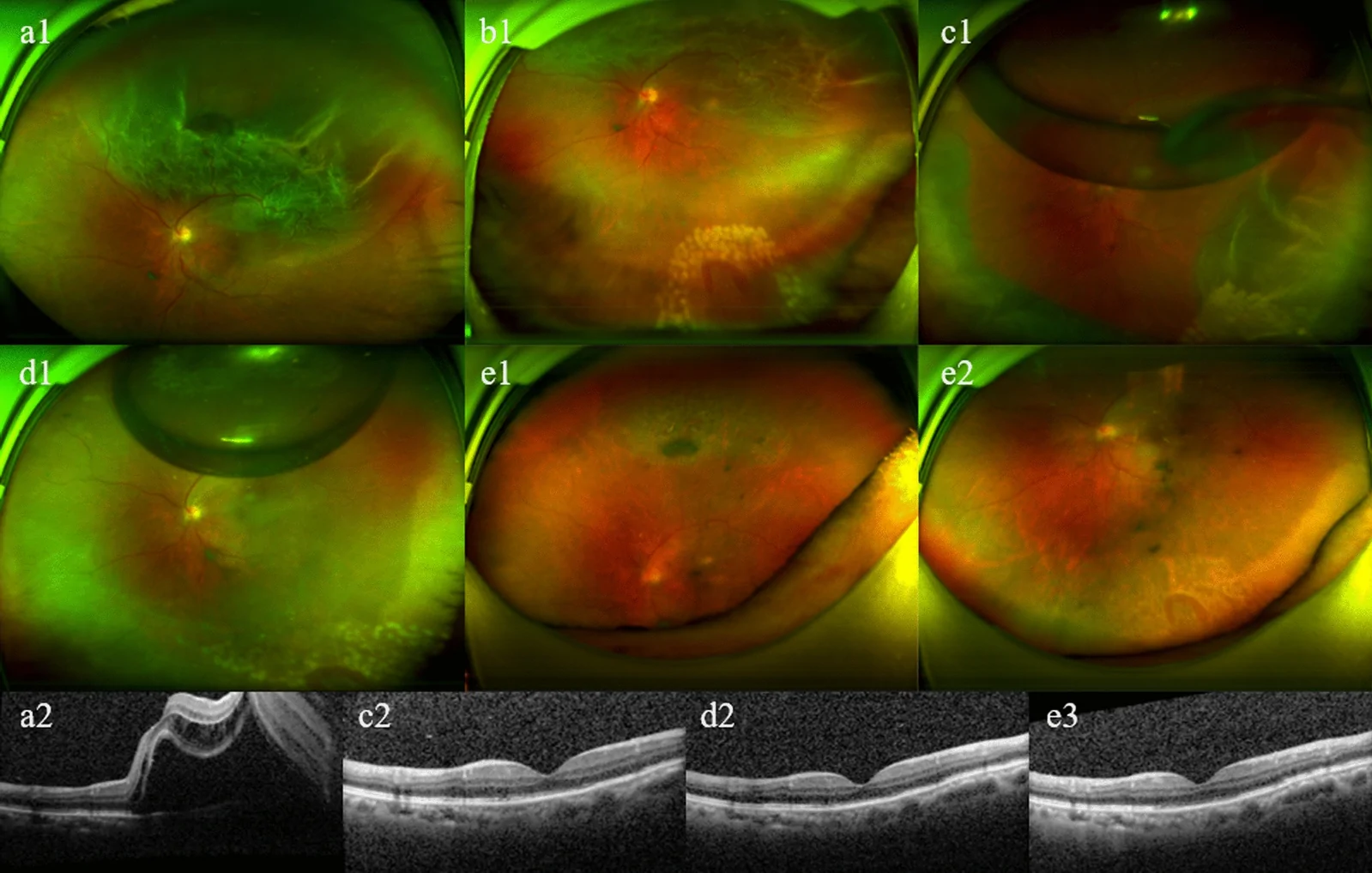
A 67-year-old man with a phakic left eye had a rhegmatogenous retinal detachment involving the macula. **a1–2** Preoperative best-corrected visual acuity (BCVA) was 20/250. Retinal detachment extended from 10 to 12 o’clock, then to 3:30, and peripheral retinal breaks were identified at 12 and 5 o’clock. **b1** Preoperative laser retinopexy had been applied around the inferior retinal break. **c1–2** On postoperative day 1, the superior retina was flat and the superior break was closed. Subretinal fluid had shifted inferiorly, resulting in inferior retinal detachment and reopening of the inferior break. Laser retinopexy was applied to the superior break, and the patient remained in a position with the superior break uppermost for 24 h. Thereafter, the patient's position was changed to elevate the inferior retinal break (prone position). **d1–2** On postoperative day 4, the inferior retina was fully attached, without visible subretinal fluid. Supplemental laser retinopexy was applied around the inferior retinal break. **e1–3** Three months postoperatively, BCVA was 20/25. The retina was fully attached and no new retinal breaks were detected

**Fig. 2:**
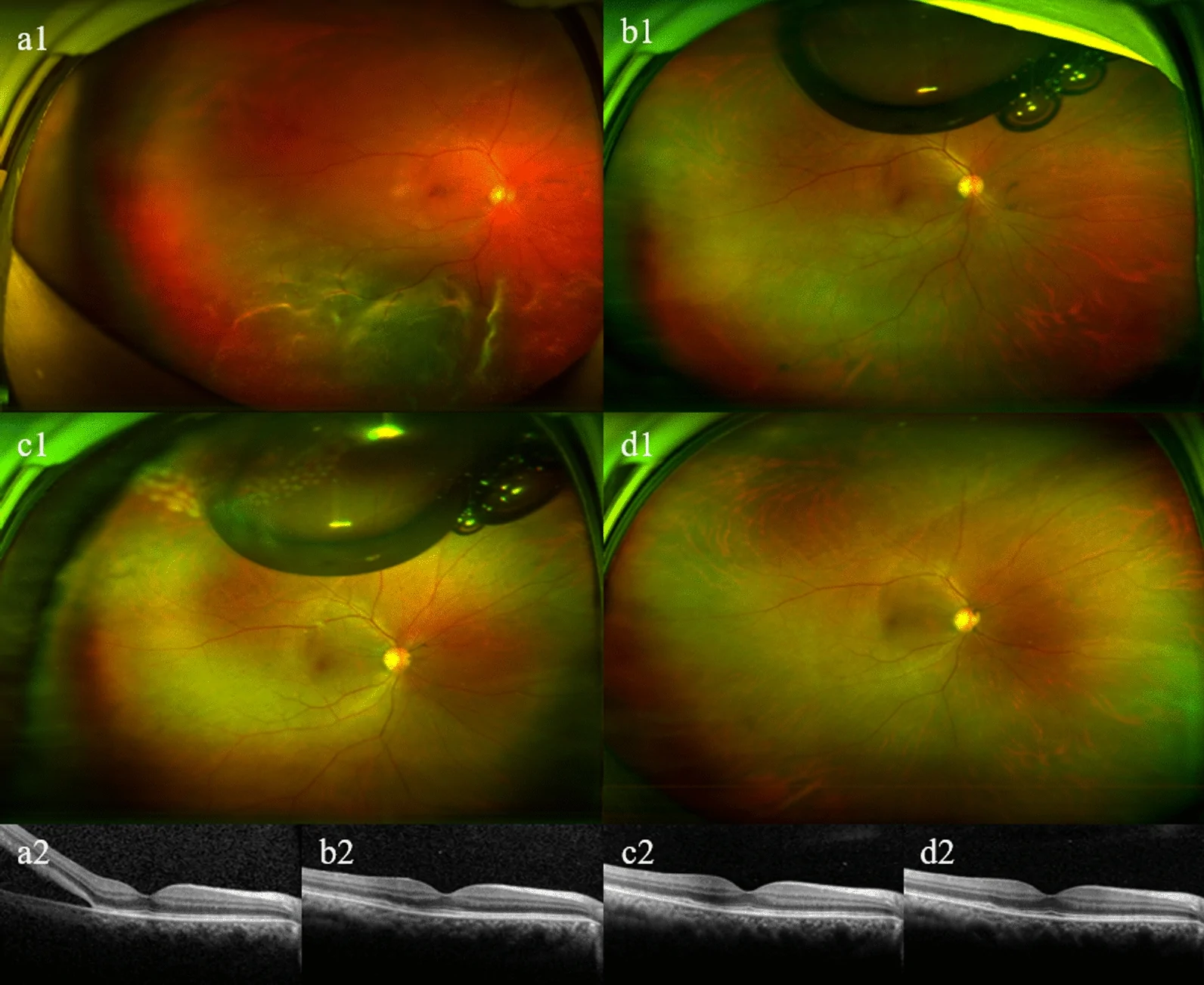
A 60-year-old woman with a phakic rhegmatogenous retinal detachment in the right eye that did not involve the macular fovea. **a1–2** Preoperative best-corrected visual acuity (BCVA) was 20/25. Retinal detachment extended from 3:30 to 12 o’clock to 1 o’clock. A peripheral retinal break is evident at 11 o’clock. **b1–2** The superior retina and posterior pole are fully attached on postoperative day 1, with a small amount of residual subretinal fluid in the inferior retina. **c1–2** Laser retinopexy around the retinal break on postoperative day 1. **d1–2** Three months postoperatively, BCVA was 20/25. The retina was fully attached and no new retinal breaks were detected

Table 1 summarizes the basic characteristics of 53 patients (53 eyes) (mean age, 43.13 ± 15.58 [range, 15–70] years; male, 28 [52.83%]; female, 25 [47.17%]). Systemic comorbidities included hypertension in 11 (20.75%) patients and type 2 diabetes mellitus in 7 (13.21%) patients. The mean axial length was 25.63 ± 1.98 (range, 21.71–29.99) mm, and 52 (98.11%) eyes were phakic. Among these, 13 and 8 eyes had mild and moderate lens opacity, respectively. Macula-off RRD was found in 20 (37.74%) eyes (Fig. 1), and 33 (62.26%) eyes had macula-on RRD (Fig. 2). The median number of retinal breaks was 1.00 (IQR, 1.00–2.00; range, 1.00–5.00), and the mean extent of retinal detachment was 2.00 ± 0.65 quadrants. The primary retinal break types were atrophic holes in 26 (49.1%) eyes, and horseshoe tears in 27 (50.9%) eyes. Retinal dialysis or giant retinal tears were not evident. The mean follow-up duration was 3.33 ± 0.84 (range, 3.00–6.37) months.

**Table 1:** Basic characteristics of patients with primary RRD treated by mPnR

Characteristic	Overall (n = 53)	Macula-off (n = 20)	Macula-on (n = 33)
Age (years)	43.13 ± 15.58	45.85 ± 15.35	41.48 ± 15.73
Sex (male/female)	28/25	13/7	15/18
Hypertension (yes/no)	11/42	6/14	5/28
Diabetes (yes/no)	7/46	2/18	5/28
Affected eye (right/left)	33/20	10/10	23/10
Axial length (mm)	25.63 ± 1.98	25.65 ± 1.75	25.62 ± 2.13
Lens status			
Clear lens	31	9	22
Mild lens opacity	13	5	8
Moderate lens opacity	8	6	2
Pseudophakic	1	0	1
Retinal detachment features			
Number of breaks	1.00 (1.00–2.00)	1.00 (1.00–2.00)	1.00 (1.00–2.00)
Extent of detachment (quadrants)	2.00 ± 0.65	2.30 ± 0.73	1.82 ± 0.53
Primary retinal break type			
Atrophic hole	26	7	19
Horseshoe retinal tear	27	13	14
Visual acuity (logMAR)			
Preoperative BCVA	0.10 (0.00–0.60)	0.75 (0.43–1.18)	0.00 (0.00–0.10)
Postoperative BCVA at 3 months	0.02 (0.00–0.20)	0.20 (0.04–0.30)	0.00 (0.00–0.10)
Last follow-up BCVA	0.02 (0.00–0.20)	0.20 (0.04–0.30)	0.00 (0.00–0.10)
Surgical outcomes			
Anatomical success at 3 months (yes/no)	51/2	20/0	31/2
Follow-up duration (months)	3.33 ± 0.84	3.24 ± 0.73	3.38 ± 0.91

*BCVA* = best-corrected visual acuity; *logMAR* = logarithm of the minimum angle of resolution; *mPnR* = modified pneumatic retinopexy; *RRD* = rhegmatogenous retinal detachment

Data are presented as mean ± standard deviation, median (interquartile range), or number, unless otherwise specified

Fluid egressed from the scleral incision intraoperatively in all 53 patients and conversion to an alternative procedure was not required. The median surgical duration was 19.00 (IQR, 16.00–22.50) minutes. The single-surgery anatomical success rate of mPnR at 3 months postoperatively was 96.23% (51/53). The median BCVA values before, and 3 months after mPnR significantly improved from 0.10 (IQR, 0.00–0.60) vs. 0.02 (IQR, 0.00–0.20) logMAR; (*P* < 0.001). None of the patients required postoperative cataract surgery at 3-months of follow-up.

Two eyes (3.77%) with macula-on RRD developed postoperative recurrent retinal detachment, which was identified at the 2-week follow-up visit. One eye (Additional File 1 Supplementary Figure S1) subsequently underwent PPV, which resulted in successful retinal reattachment and BCVA recovery to 20/20 at 3 months postoperatively. The other eye (Additional File 1 Supplementary Figure S2) eventually underwent PPV elsewhere, and telephone follow-up at 3.5 months confirmed reattachment with a BCVA of 20/25. However further visits declined and data were lost.

One patient (1.89%) with macula-on RRD was diagnosed with a secondary epiretinal membrane at the 3-month follow-up (Additional File 1 Supplementary Figure S3). The BCVA decreased from 20/32 to 20/100. Subsequently, the patient underwent PPV, phacoemulsification with intraocular lens implantation, epiretinal membrane peeling, and internally limiting membrane peeling. The BCVA improved to 20/32 at 3 months after the combined procedure. Two eyes developed small, non-clinically significant subretinal hemorrhages adjacent to the drainage paracentesis site, which were detected on postoperative day 1 (Additional File 1 Supplementary Figures S4 and S5). Ten and 43 (81.13%) surgeries proceeded in an inpatient operating room and our standard outpatient ophthalmic surgical suite, respectively. Assignment to outpatient or inpatient operating suites was determined simply based on their availability and nursing schedules and not by case complexity or clinical criteria.

### Subgroup analysis: macula-off RRD

**Fig. 3:**
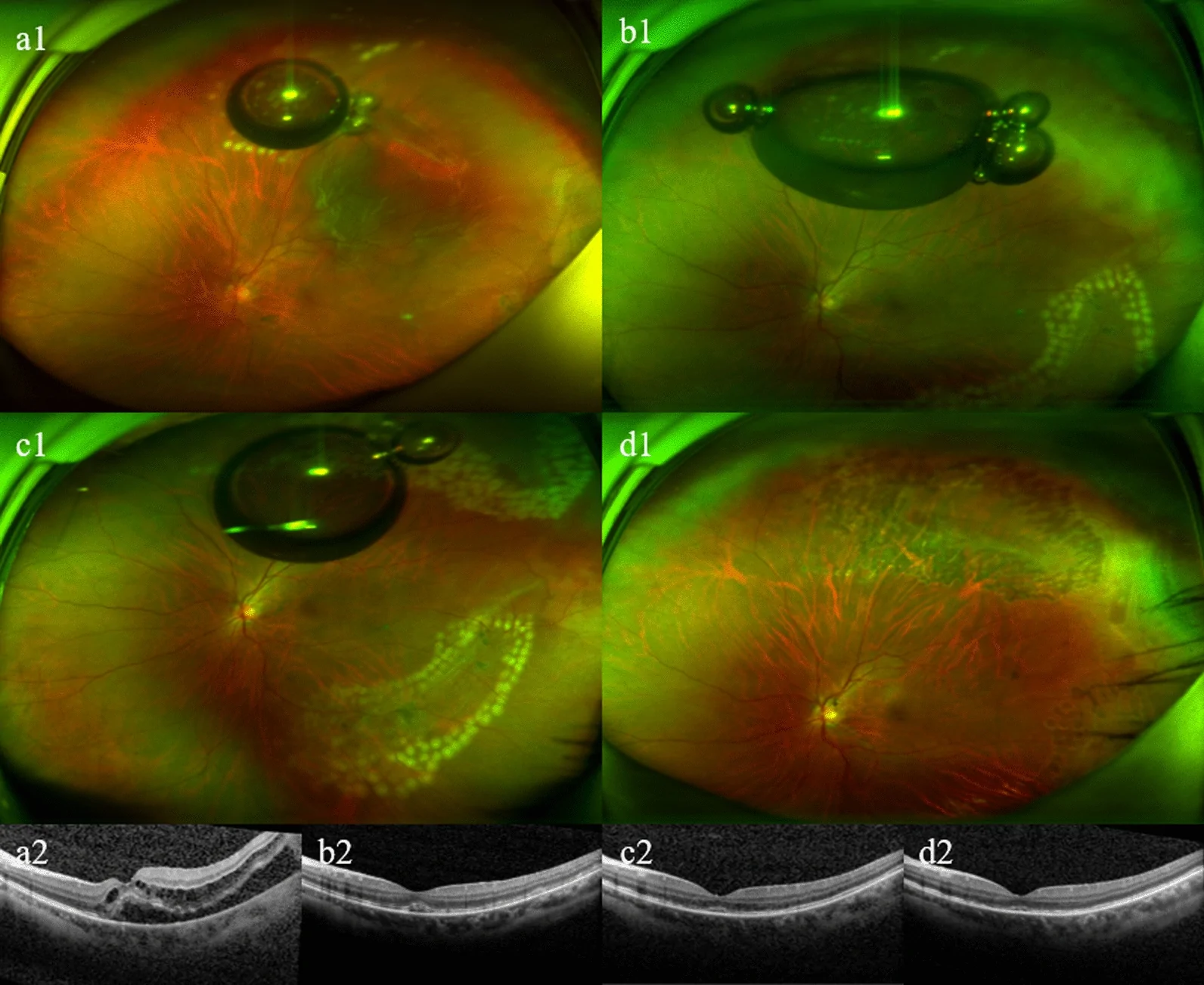
A 50-year-old man with phakic rhegmatogenous retinal detachment involving the macula in the left eye. Pneumatic retinopexy applied elsewhere one week before had failed to reattach the retina. **a1–2** Preoperative BCVA was 20/50. Retinal detachment extended from 0:30 to 3:30, and retinal breaks were identified at the edge of a degenerative band at 1:30 and 6 o’clock. **b1–2** Subretinal fluid was absent on postoperative day 1, and the retina was fully attached. Laser retinopexy was applied around the retinal breaks and the degenerative band. **c1–2** The retina remains fully attached on postoperative day 2. Laser retinopexy was completed around the breaks and degenerative band. **d1–2** The BCVA was 20/20 at 4 months postoperatively. The retina was fully attached and no new retinal breaks were detected

A macula-off subgroup included 20 eyes in 20 patients (mean age, 45.85 ± 15.35 years; range, 19–67 years) and 13 (65.00%) were male. The mean axial length was 25.65 ± 1.75 (range, 23.24–29.06) mm. All eyes were phakic, including five with mild lens opacity and six with moderate lens opacity. The median number of retinal breaks was 1.00 (IQR, 1.00–2.00; range, 1.00–4.00), and the mean extent of retinal detachment was 2.30 ± 0.73 quadrants. The single-surgery anatomical success rate at 3 months was 100% (20/20) in this subgroup, which did not significantly differ from that of the macula-on subgroup (93.9% [31/33]; *P* = 0.521). The median BCVA significantly improved from preoperative 0.75 (IQR, 0.43–1.18) logMAR to postoperative 0.20 (IQR, 0.04–0.30) logMAR (*P* < 0.001). Retinal reattachment was not achieved in two patients with macula-off RRD who had been treated by conventional PnR elsewhere one week earlier. However, retinal reattachment was complete in both eyes after mPnR. The BCVA improved to 20/50 at 3 months in one eye, and 20/20 in the other at 4 months postoperatively (Fig. 3).

## Discussion

We evaluated the ability of an mPnR innovation that combines transscleral drainage of SRF with intravitreal air injection to treat primary RRD. The conceptual origins of this approach date back to the early twentieth century. In 1911, Ohm described a method involving intravitreal air injection after SRF drainage through a paracentesis for the treatment of RRD [15]. However, this technique primarily relied on patient positioning to maximize contact between the air bubble and the detached retina. It also lacked a clear understanding of the underlying pathophysiology of retinal detachment. Although the single reported case was successful, it is now believed to have involved a substantial element of chance [16]. Subsequently, intravitreal air injection was combined with diathermy to treat RRD [17]. In this method, intraocular air mainly served as a vitreous expander to maintain intraocular pressure and facilitate external SRF drainage, rather than as a direct tamponade of retinal breaks. A major advance was made in 1938 when Rosengren introduced the concept of retinal break “tamponade” [18]. He applied diathermy strictly to the area corresponding to a retinal break, drained the SRF, and injected an air bubble specifically to tamponade the break. This method achieved a reattachment rate of 77%, so a substantial proportion of patients experienced redetachment. This outcome was likely related to the premature absorption of air bubbles before the formation of a sufficiently strong chorioretinal adhesion around the break. The technical limitations of the era, including imprecise localization of retinal breaks and difficulty restricting diathermy application to the immediate surrounding area, might also have contributed [19]. Because of the subsequent development and widespread adoption of SB, PnR, and PPV, further evolution of RRD repair techniques combining air injection with SRF drainage has largely stalled. Numerous studies have demonstrated that PnR achieves single-surgery success rates comparable to PPV and SB for RRD, with potentially superior postoperative visual recovery [9–13, 20]. However, conventional PnR remains limited by needs for prolonged strict facial positioning and expansile gas. The mPnR technique was developed as an advancement of the conventional PnR, and its integration of air injection with SRF drainage has overcome these limitations. In the modern era, high-resolution wide-field imaging enables precise localization of retinal breaks, whereas contemporary laser photocoagulation permits accurate and rapid induction of chorioretinal adhesions. Consequently, most of the historical limitations constraining the effectiveness of this approach have now been largely resolved.

We achieved a single-surgery retinal reattachment rate of 96.23% after mPnR to treat primary RRD. The rate was notably 100% in the macula-off subgroup, which was analyzed specifically because this represents the most clinically challenging scenario, in which delayed reattachment leads to the risk of irreversible photoreceptor damage. However, given the small subgroup and the absence of statistical significance in the between-group comparison (macula-on 93.9% vs. macula-off 100%, *P* = 0.521), our results should be interpreted cautiously and validated in larger studies. For contextual reference only, previous studies have reported success rates of approximately 93.2% for PPV and 82% for SB in comparable RRD populations [9, 11]. While these outcomes appear encouraging, we caution that direct comparison with these historical benchmarks is precluded by differences in study design, patient selection, and surgical expertise.

Furthermore, substantial functional improvement was achieved, as the preoperative median BCVA improved from 0.10 to 0.02 logMAR at 3 months postoperatively (*P* < 0.001). Subgroup analysis of visual recovery among patients with macula-off RRD was even more pronounced, as the BCVA improved from 0.75 (preoperative) to 0.20 logMAR; (*P* < 0.001). Collectively, these findings support the anatomical and functional ability of mPnR to treat primary RRD.

The rate of SRF resolution adjacent to the retinal break is a critical determinant of PnR success. However, the absorptive capacity of the retinal pigment epithelium substantially varies among individuals [21–23]. The “steam-roller” maneuver, which uses the buoyancy of an intravitreal gas bubble to displace SRF through the retinal break, can accelerate reattachment. However, its effectiveness is often limited by small retinal breaks or highly viscous SRF. Furthermore, the SRF clearance rate directly determines the duration of postoperative positioning. Conventional PnR typically requires maintaining a specific position for at least 5 days up to 3 weeks, which imposes a considerable burden on patient compliance and quality of life.

In contrast, mPnR incorporates deliberate SRF drainage through paracentesis, which accelerates retinal reattachment, and creates an additional intraocular space for injecting more air. This provides a more substantial tamponade and facilitates further evacuation of the residual SRF through the paracentesis site, thereby promoting rapid repositioning of the neurosensory retina to the retinal pigment epithelium. A larger air bubble offers a more effective and sustained tamponade without requiring expansile gas. Concurrently, it reduces the stringency of postoperative positioning requirements, because patients only need to maintain a position that allows the bubble to cover the retinal break. In this series, all patients underwent laser photocoagulation within 24 h of mPnR. Consequently, the required postoperative positioning duration was reduced to 3–5 days, potentially constituting an advance over conventional PnR. This modification significantly reduces the burden of patient compliance and minimizes the disruption of daily activities, which might facilitate successful retinal reattachment. However, the high single-surgery success rate achieved herein likely reflects, at least in part, stringent patient selection rather than solely the technical advantages of mPnR. We applied the PIVOT trial criteria, which inherently selects patients that are suitable for PnR. The range of success rates after PnR is 81%–90% in selected populations [11, 24]. Independent validation of this success rate in a prospective, multicenter study with a concurrent control arm is needed before any definitive conclusions can be drawn about the advantages of mPnR.

Post hoc analyses of the PIVOT trial revealed that conventional PnR relies on slow SRF absorption via the retinal pigment epithelium pump, which is a physiological reattachment process that might protect visual quality. Muni et al. found significantly lower rates of ellipsoid zone and external limiting membrane discontinuity after PnR vs. PPV at 12 months (7% vs. 24% and 6% vs. 20%), suggesting slower reattachment reduces photoreceptor damage [25]. Lee et al. showed outer retinal folds were more frequent after PPV than PnR (34.1% vs. 14.3%) and correlated with worse 1-year vision [12]. This indicated that rapid SRF drainage increases fold risk by preventing the resolution of outer retinal corrugations. Bansal et al. also found that early persistent subfoveal fluid transiently reduced vision but recovered with resorption, underscoring the metabolic benefits of gradual absorption [26]. Thus, while active SRF drainage in mPnR might facilitate faster anatomic reattachment, it might also shift the technique toward rapid reattachment, with potential implications for photoreceptor integrity and outer retinal fold formation. Future prospective randomized trials using SD-OCT and en-face OCT to compare mPnR and PnR are needed to clarify the long-term visual impact of accelerated SRF drainage and to explore optimization strategies for this technique.

Furthermore, PPV and SB must proceed in an operating room under sedation and retrobulbar or peribulbar anesthesia. They both require specialized surgical equipment and consumables. In contrast, mPnR, similar to conventional PnR, can proceed under subconjunctival anesthesia alone, without the need for sedation or regional blocks, which reduces anesthesia-related risk. A brief and efficient procedure can be completed without relying on an operating microscope or a binocular indirect ophthalmoscope, making it suitable for an outpatient setting. Both mPnR and conventional PnR offer the advantages of high efficiency, minimal invasiveness, and low healthcare costs. The principal difference is that mPnR replaces anterior chamber paracentesis with transscleral paracentesis for SRF drainage. Accordingly, mPnR might be regarded as an evolution of the PnR technique and can be considered as a potential alternative treatment option for patients with RRD that meet the PIVOT trial criteria. The mPnR technique is particularly valuable in remote or resource-limited regions (where surgical resources are scarce and intraocular gas is often unavailable), because it provides a practical and timely option for RRD management.

Paracentesis for SRF drainage should occur at the site of maximal retinal elevation adjacent to the retinal break(s). An oblique incision is recommended to reduce the risk of an iatrogenic retinal break. If such a break occurs, its proximity to the primary break allows concurrent treatment during subsequent laser photocoagulation. An additional potential benefit might be achieved for eyes with a sufficiently large retinal break, because a temporary fluid pathway might form between the vitreous cavity and the paracentesis site through it, thus permitting the egress of intravitreal fluid. In eyes with shallow SRF, where the limited subretinal space makes passive external drainage difficult, placing paracentesis immediately adjacent to the break is particularly critical. This outflow reduces the intraocular volume, which creates additional space for air injection and facilitating retinal reattachment, even when the SRF is minimal. Moreover, such outflow might facilitate the removal of dispersed retinal pigment epithelial cells and other potential mediators from the vitreous cavity, potentially contributing to the reduction of intraocular factors associated with proliferative vitreoretinopathy [27]. Nevertheless, eyes with shallow SRF remain susceptible to failed drainage of SRF. Should external drainage prove to be completely unsuccessful intraoperatively, particularly in the absence of fluid egress, conversion to conventional PnR or SB should be considered.

However, although mPnR offers the advantages of avoiding expansile gas and reducing the burden of face positioning, transscleral drainage paracentesis introduces risks that do not exist in conventional PnR such as subretinal hemorrhage and vitreous incarceration with potential vitreoretinal traction [28]. Only two eyes (3.77%) in our series developed small, clinically insignificant subretinal hemorrhages adjacent to the paracentesis site. We attributed this favorable safety profile to continuous scleral indentation combined with immediate intraocular air injection, which maintains stable intraocular pressure throughout the procedure and minimizes the risk of hemorrhage. Vitreous incarceration with vitreoretinal traction did not occur. Nevertheless, the potential risk of vitreous incarceration cannot be overlooked, and subtle vitreoretinal traction might prevent clinical detection. This risk is an important focus for future studies.

Conversely, the mPnR eliminates certain risks inherent in conventional PnR. Although conventional PnR might require anterior chamber paracentesis, the mPnR technique eliminates this step, thereby avoiding the associated risk of iatrogenic angle-closure glaucoma caused by anterior chamber shallowing [29].

The retina surrounding the break is reattached at the conclusion of surgery in most mPnR procedures. However, because patients are often stressed and frequently exhibit corneal epithelial edema on the day of surgery, it is advisable to defer laser photocoagulation for 12–24 h after surgery. However, intraocular air bubbles complicate laser applications. Depending on the location of the retinal break and the size of the air bubble, the following approaches can be applied: rotate the patient’s head to expose the retinal break for laser treatment, or, if the bubble is sufficiently large, position the break at the center of the bubble to allow laser application through it. Cryotherapy applied to a retinal break area before air injection might be a viable alternative. Algvere et al. applied a combination of cryotherapy, SRF drainage, and air injection [30]. However, the primary retinal reattachment rate of 86% was notably lower than our findings. This disparity is likely attributable to the fact that chorioretinal adhesion typically develops more slowly after cryotherapy than after laser photocoagulation [31, 32]. Because the mPnR technique uses air, which is absorbed relatively quickly, more rapid adhesion achieved with laser photocoagulation might be more appropriate in this setting.

Two eyes in this series with macula-off RRD that persistently detached after PnR became completely reattached after a subsequent mPnR. This outcome suggests that mPnR could be a treatment option for selected patients with failed initial PnR, provided that their clinical features remain appropriate for the mPnR approach. Another patient experienced an unusual sequence of events (Fig. 1). We reattached a superior retinal break after mPnR, but residual SRF shifted inferiorly, which caused an extant retinal break to reopen at the 5 o’clock position that resulted in secondary inferior RRD. We immediately laser photocoagulated the superior break while maintaining the uppermost position. We changed the position of the patient 24 h later to a head-low, prone posture to allow the air–fluid interface to cover the inferior break. These measures resulted in complete retinal reattachment. Therefore, we inferred that mPnR could be applied to RRDs with breaks in the inferior quadrant (from 4 to 8 o’clock). However, this would be premature and overly speculative. Nonetheless, this successful outcome offers a potential avenue for future exploration.

This study had several limitations. The retrospective design and relatively small sample limited the generalizability of our findings. The minimum follow-up was only 3 months. Although the PIVOT trial uncovered a low likelihood of repeated detachment beyond 3 months post-PnR, a longer follow-up is required to fully assess subjective visual outcomes, long-term complications such as epiretinal membrane formation, and the incidence of late repeated detachment. We did not directly compare mPnR and other retinal detachment repair techniques, such as conventional PnR, PPV, or SB. Prospective controlled studies are required to address these limitations.

## Conclusion

Preliminary safety and efficacy profiles for treating primary RRD using mPnR were favorable, in eyes that met the PIVOT trial criteria. Active SRF drainage accelerated retinal reattachment and reduced the requirement for burdensome postoperative positioning. The single-operation, anatomical success rate was 96.23%. The modified PnR is efficient, minimally invasive, and does not require expansile gas. It can be implemented in an outpatient operating suite, and offers a simplified, less invasive surgical option for eligible patients.

## Supplementary Information

Additional File 1. Supplementary Figures

Additional File 2. Supplementary Video. Modified pneumatic retinopexy with transscleral subretinal fluid drainage in a patient with primary rhegmatogenous retinal detachment. Key procedural steps are subconjunctival anesthesia, placement of traction sutures on the rectus muscles adjacent to the retinal break, localized conjunctival incision, transscleral subretinal fluid drainage, intravitreal air injection, and conjunctival incision closure using electrocoagulation

## Data Availability

The datasets generated and/or analyzed in the current study are available from the corresponding author upon reasonable request.

## Abbreviations

BCVA: Best-corrected visual acuity
IQR: Interquartile range
LogMAR: Logarithm of the minimum angle of resolution
mPnR: Modified pneumatic retinopexy
PIVOT: Pneumatic Retinopexy versus Vitrectomy for the Management of Primary Rhegmatogenous Retinal Detachment
PnR: Pneumatic retinopexy
PPV: Pars plana vitrectomy
RRD: Rhegmatogenous retinal detachment
SB: Scleral buckling
SRF: Subretinal fluid
