# Supplementary material for: Outcomes of modified pneumatic retinopexy with transscleral subretinal fluid drainage for primary rhegmatogenous retinal detachment
Source: Eye Vis (Lond). 2026 Aug 26;13:37. doi: 10.1186/s40662-026-00506-z (PMC13508407; doi:10.1186/s40662-026-00506-z)
Supplement: Supplementary file 1 [file 40662_2026_506_MOESM1_ESM.docx]

**Additional file 1 Supplementary figures**

**Supplementary Figure S1.** A 43-year-old male with phakic macula-on rhegmatogenous retinal detachment (RRD) in the right eye. The patient was subsequently treated with pars plana vitrectomy (PPV) after unsuccessful modified pneumatic retinopexy (mPnR). **a1–2** Preoperative imaging of macula-on RRD. **b1–2** One day 1 after mPnR with retinal laser photocoagulation. **c1–2** New superior retinal break with adjacent shallow detachment at 14 days after mPnR. **d1–2** Retina is attached one month after PPV. **e1–2** Final follow-up image after PPV, confirming sustained anatomical attachment.

**Supplementary Figure S2.** A 38-year-old phakic female with macula-on rhegmatogenous retinal detachment (RRD) in the left eye. The patient subsequently underwent pars plana vitrectomy elsewhere after modified pneumatic retinopexy (mPnR) failed. **a1–2** Preoperative imaging of macula-on RRD. **b1-2** Modified mPnR and subsequent laser photocoagulation on postoperative day 1. **c1–2** Recurrent retinal detachment at two weeks postoperatively. **d1–2** Immediate postoperative image after second mPnR. **e1–2** Image shows laser photocoagulation after second mPnR. **f1–2** Final follow-up image before secondary vitrectomy elsewhere reveals recurrent retinal detachment (arrows).

**Supplementary Figure S3.** A 62-year-old female with phakic, right-eye macula-on rhegmatogenous retinal detachment (RRD). The patient was treated with modified pneumatic retinopexy (mPnR) subsequently underwent pars plana vitrectomy (PPV) for a secondary epiretinal membrane. **a1–2** Preoperative image of macula-on RRD. **b1–2** Postoperative day 1. **c1–2** Post-laser photocoagulation image shows demarcated retinal breaks. **d1–2** Three-month follow-up image reveals a secondary epiretinal membrane with macular involvement. **e1–2** One week after combined PPV, phacoemulsification with intraocular lens implantation, and epiretinal/internal limiting membrane peel. **f1–2** Final follow-up image at 3 months after these operations shows anatomical restoration and improved macular contour.

**Supplementary Figure S4.** Phakic macula-off rhegmatogenous retinal detachment (RRD) complicated by a small, non-clinically significant subretinal hemorrhage in the left eye of a 24-year-old female after modified pneumatic retinopexy (mPnR). **a1–2** Preoperative image of RRD before mPnR. **b1–2** Image after mPnR on postoperative day 1, shows a small, non-clinically significant subretinal hemorrhage adjacent to the paracentesis drainage site (arrow). **c1–2** Post-laser photocoagulation image shows confluent laser scars surrounding the retinal break. **d1–2** Final follow-up image shows complete resolution of the subretinal hemorrhage and attached retina.

**Supplementary Figure S5.** A 24-year-old female with phakic macula-on rhegmatogenous retinal detachment (RRD) in the right eye complicated by a small, non-clinically significant subretinal hemorrhage after modified pneumatic retinopexy (mPnR). **a1–2** Preoperative image of RRD before mPnR. **b1–2** Image after mPnR on postoperative day 1 shows a small, non- clinically significant subretinal hemorrhage adjacent to the paracentesis drainage site (arrow). **c1–2** Post-laser photocoagulation image shows confluent laser scars surrounding the retinal break. **d1–2** Final follow-up image shows complete resolution of the subretinal hemorrhage and attached retina.
